# Le méningiome intra osseux: à propos de deux cas

**DOI:** 10.11604/pamj.2018.30.220.15364

**Published:** 2018-07-20

**Authors:** Siham Nasri, Zineb Marzouki, Imane Kamaoui, Imane Skiker

**Affiliations:** 1 Service de Radiologie CHU Mohammed VI, Oujda, Maroc

**Keywords:** Méningiomes intra osseux, TDM, IRM, Intrabone meningiomas, CT scan, MRI

## Abstract

Les méningiomes intra osseux primitifs sont des méningiomes ectopiques rares, qui constituent 1 à 2% de tous les méningiomes. Le but de ce travail est d'illustrer les différents aspects à l'imagerie contribuant à une approche diagnostique et de discuter leurs différents diagnostics différentiels. Nous rapportons, à ce propos, deux cas de méningiomes intra osseux à localisation ptérionale, documentés par TDM et IRM.

## Abstract (en)

Primary intrabone meningiomas are rare ectopic meningiomas accounting for 1- 2% of all meningiomas. This study aims to illustrate imaging characteristics of intrabone meningiomas contributing to diagnosis and to discuss their differential diagnoses. We report two cases of pterional intrabone meningiomas detected using CT scan and MRI.

## Introduction

Les méningiomes encéphaliques représentent 20% des tumeurs intra cérébrales primitives et sont habituellement considérées comme des lésions bénignes. Les localisations intra-osseuses sont rares et constituent seulement 1 à 2% de l'ensemble des méningiomes intracrâniens. Nous rapportons deux cas de méningiomes intra-osseux sphéno- temporo-orbitaires documentés par TDM et IRM.

## Patient et observation

**Figure 1:**
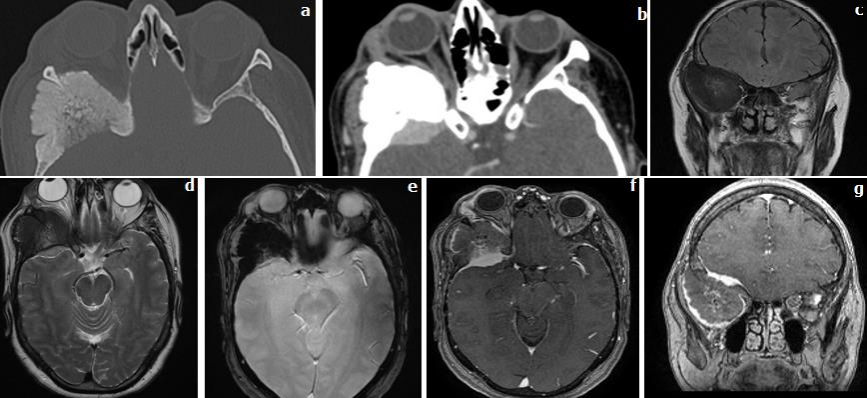
Coupes axiales scannographiques en fenêtre osseuse (a) et parenchymateuse (b) après injection du produit de contraste iodé; IRM en séquence coronale Flair (c), axiale T2(d) et T2(e), axiale et coronale T1 GADO+ (f,g)

**Observation 1:** Patiente âgée de 56 ans ayant comme antécédent un traumatisme crânien il y a 10 ans, et qui présente une exophtalmie droite progressive et douloureuse évoluant depuis 8 ans et s'accompagnant d'une déformation de la région temporale droite (Figure 1).

**Figure 2:**
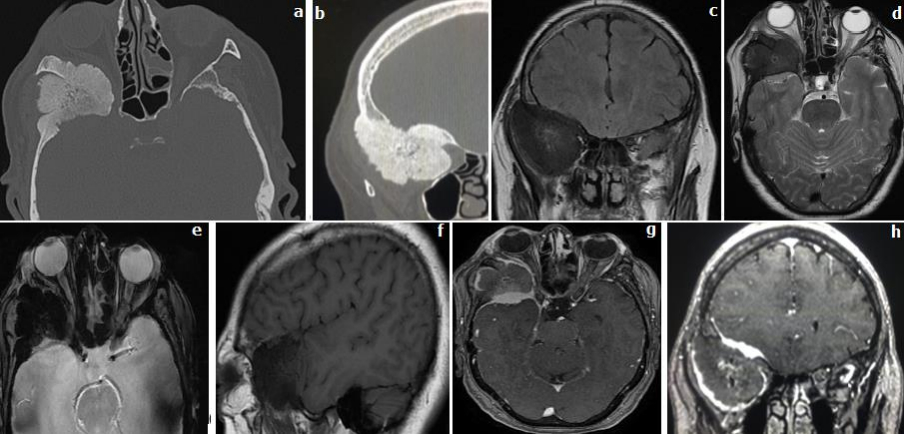
TDM en coupe axiale (a) et reconstruction coronale (b) en fenêtre osseuse; IRM en séquence coronale Flair (c), axiale T2 (d) et T2(e), sagittale T1 (f) et axiale et coronale T1 GADO+ (g,h)

**Observation 2:** Une femme de 56 ans, sans antécédents pathologiques notables, qui consulte pour une exophtalmie et une tuméfaction périorbitaire droite (Figure 2). Dans les deux observations la TDM a objectivé un épaississement et condensation du processus sphéno-temporo-orbitaire droit avec dédifférenciation cortico-spongieuse (tables-diploé) et aspect spiculé des tables interne et externe, associé à un petit épaississement et rehaussement méningé en regard de l'os condensé. L'IRM a confirmé le processus lésionnel intra osseux ptérional droit en hyposignal sur toutes les séquences, associé à un rehaussement des méninges adjacentes après injection du Gadolinium. A noter le rehaussement central intra lésionnel.

## Discussion

Les méningiomes sont les tumeurs primitives intra crâniennes non gliales les plus fréquentes, le plus souvent situées dans l'espace sous dural [1]. Les méningiomes ectopiques peuvent siéger au niveau des sinus de la face, de la cavité nasale, des orbites, des glandes salivaires, du nasopharynx et de la voûte crânienne. Les méningiomes intra osseux primitifs sont rares et ils constituent une entité distincte. Leur premier cas a été décrit par Winkler en 1904, la convexité et la base du crâne étant les localisations les plus fréquentes [2].

**Physiopathologie:** Leur physiopathologie est mal connue et plusieurs hypothèses ont été avancées en ce sens: La séquestration des cellules arachnoïdiennes au niveau des traits de fractures après traumatisme crânien [2]. La prolifération de cellules arachnoïdiennes ectopiques. La prolifération de reliquats de cellules arachnoïdiennes extra-durales situées au niveau des sutures crâniennes lors de l'accouchement et du remodelage crânien. Le développement à partir de cellules mésenchymateuses pluripotentes ou à partir de la métaplasie des cellules mésenchymateuses telles que les fibroblastes [3].

**Clinique:** La symptomatologie clinique n'est pas spécifique. Les manifestations varient en fonction du siège de la lésion, sa taille et les rapports avec les organes de voisinage [2]. Le plus souvent ces lésions se manifestent par une tuméfaction fixe, dure et indolore. L'examen neurologique est normal en dehors de céphalées intermittentes. Dans le cas de tumeurs s'étendant à la table interne et entrainant un effet de masse sur le parenchyme cérébral, on peut observer des signes neurologiques. Dans les formes temporo sphéno-orbitaires, la clinique est dominée par une exophtalmie lentement progressive axile, indolore, associée à une tuméfaction de la fosse temporale homolatérale. La baisse de l'acuité visuelle est variable [1, 2].

### Moyens d'exploration

En radiographie standard: le méningiome intra osseux peut prendre deux formes: la forme dite hyperostosante est la plus fréquente avec épaississement de la voûte, hérissée parfois de spicules osseux en « rayon de soleil », et bien visible sur les clichés en incidence tangentielle, et la forme ostéolytique ou érosive, avec lyse osseuse associée ou non à une reconstruction. Des calcifications intratumorales peuvent exister dans 20% des cas.

L'examen tomodensitométrique: permet de découvrir le méningiome qui a une densité tissulaire, le plus souvent supérieure à celle du cortex. Dans la forme hyperostosante, la TDM permet d'apprécier l'aspect trapu et régulièrement disposé des spicules, différent de l'aspect fin et anarchique d'une tumeur maligne.

L'imagerie par résonance magnétique: permet une meilleure définition anatomique en montrant ses différentes composantes. La tumeur se traduit par un hyposignal marqué sur les séquences pondérées en T1 et en T2. L'injection intraveineuse de gadolinium entraîne une prise de contraste au sein de la composante intra-osseuse et au niveau des enveloppes méningées en regard. Elle permet un bilan d'extension méningé qui est souvent sous-estimé par le scanner du fait de son faible contraste différentiel entre l'os et les méninges rehaussées permettant ainsi d'éviter une récidive par exérèse partielle. L'IRM permet également de détecter une extension intracrânienne [4].

La scintigraphie au technétium: Montre une hyperfixation dans la majorité des cas [5].

Diagnostics differentiels: Dans les formes hyperostosantes, ils se discutent avec les hémangiomes sclérotiques (lésion bien limitée, trabéculaire en rayon de miel, réaction périostée, hypersignal T1 hétérogène en IRM), les dysplasies fibreuses (aspect en verre dépoli, contours nets), l'ostéome (contours réguliers et nets), les métastases ostéoblastiques et la maladie de Paget. Pour les formes lytiques, le diagnostic différentiel peut se poser avec les métastases ostéolytiques, l'ostéosarcome (spicules fins et anarchiques, alors que dans les MIO, ils sont trapus et réguliers) et la lacune myélomateuse. Chez le sujet jeune, il faudra discuter en plus les granulomes éosinophiles et les kystes épidermoîdes [1, 2].

Traitement: Le traitement est chirurgical et consiste en une exérèse qui permet de poser le diagnostic histologique et d'éviter les complications neurologiques. Une radiothérapie complémentaire peut être préconisée dans les cas d'exérèse incomplète [6].

## Conclusion

Les méningiomes intra osseux sont des tumeurs ectopiques rares, bénignes et d'évolution progressive. Leur aspect en imagerie est caractéristique avec très bonne étude topographique. Le diagnostic de certitude est histologique.

## Conflits d’intérêts

Les auteurs déclarent aucun conflits d'intérêts.

## References

[1] Henon A, Colombat M, Rodallec M, Redondo A, Feydy A (2005). Méningiome intra-osseux de la voûte du crâne: confrontation anatomo-radiologique. Journal de Radiologie.

[2] Benchakroun F, Ech-Cherif El Kettani N, Arkha Y, Chakir N, El Khamlichi A, El Hassani M R, Jiddane M (2011). Le méningiome intra-osseux sphénotemporo-orbitaire. Feuillets de radiologie.

[3] Jan M, Velut S, Lefrancq T (1993). Méningiomes intracrâniens. Encyclopédie médico-chirurgicale (Paris).

[4] Elkharras A, En-Nouali H, Jawhari N, Elhaddad A, Ajja A, Chaouir S, Benamaur M (2005). Méningiome géant a développement intra et extra crânien. African journal of neurological sciences.

[5] Ridouh M, Zemallache Megueni A, Krim M, Mahida B, Berber N (2015). Méningiome intra-osseux: cause de faux positifs dans la recherche de métastases osseuses par scintigraphie aux biphosphonates. Revue Neurologique.

[6] Dietemann J L, Abu Eid M, Mourao Soares I, Bogorin A, Boyer P, Draghici S (2012). Tumeurs cranioencéphaliques: tumeurs extra-axiales. Neuro-imagerie diagnostique.

